# Quality of life and its predictors in adults with tuberous sclerosis complex (TSC): a multicentre cohort study from Germany

**DOI:** 10.1186/s42466-021-00130-3

**Published:** 2021-06-28

**Authors:** Johann Philipp Zöllner, Nadine Conradi, Matthias Sauter, Markus Knuf, Susanne Knake, Gerhard Kurlemann, Thomas Mayer, Christoph Hertzberg, Astrid Bertsche, Ilka Immisch, Karl Martin Klein, Klaus Marquard, Sascha Meyer, Anna H. Noda, Felix von Podewils, Hannah Schäfer, Charlotte Thiels, Bianca Zukunft, Susanne Schubert-Bast, Janina Grau, Laurent M. Willems, Felix Rosenow, Jens-Peter Reese, Adam Strzelczyk

**Affiliations:** 1 Epilepsy Center Frankfurt Rhine-Main, Center of Neurology and Neurosurgery, Goethe-University Frankfurt, Schleusenweg 2-16 (Haus 95), 60528 Frankfurt am Main, Germany; 2 Center for Personalized Translational Epilepsy Research (CePTER), Goethe-University Frankfurt, Frankfurt am Main, Germany; 3 Klinikum Kempten, Klinikverbund Allgäu, Kempten/Allgäu, Germany; 4 Department of Pediatrics, Klinikum Worms, Worms, Germany; 5 Department of Pediatrics, University Medicine Mainz, Mainz, Germany; 6 Epilepsy Center Hessen and Department of Neurology, Philipps-University Marburg, Marburg (Lahn), Germany; 7 St. Bonifatius Hospital, Lingen, Germany; 8 Epilepsy Center Kleinwachau, Radeberg, Germany; 9 Department of Neuropediatrics, Vivantes Klinikum Neukölln, Berlin, Germany; 10 Department of Neuropediatrics, University Hospital for Children and Adolescents, Rostock, Germany; 11 Departments of Clinical Neurosciences, Medical Genetics, and Community Health Sciences, Hotchkiss Brain Institute & Alberta Children’s Hospital Research Institute, Cumming School of Medicine, University of Calgary, Calgary, Alberta Canada; 12 Department of Pediatric Neurology, Psychosomatics and Pain Management, Klinikum Stuttgart, Stuttgart, Germany; 13 Department of Neuropediatrics, University Children’s Hospital of Saarland, Homburg, Germany; 14 Department of Neurology, Epilepsy Center, University Medicine Greifswald, Greifswald, Germany; 15 Division of Nephrology, Medizinische Klinik und Poliklinik IV, Klinikum der LMU München – Innenstadt, Munich, Germany; 16 Department of Nephrology, Klinikum rechts der Isar, Technische Universität München, Munich, Germany; 17 Department of Neuropediatrics and Social Pediatrics, Ruhr University Bochum, Bochum, Germany; 18 Department of Nephrology and Internal Intensive Care, Charité - University Medicine Berlin, Berlin, Germany; 19 Department of Neuropediatrics, Goethe-University Frankfurt, Frankfurt am Main, Germany; 20 Institute of Clinical Epidemiology and Biometry, University of Würzburg, Würzburg, Germany

**Keywords:** TSC, Tuberous sclerosis complex, Seizure, Epilepsy, Organ manifestations, Quality of life, Rare disease, EQ-5D, QOLIE-31, mTOR inhibitor

## Abstract

**Background:**

Tuberous sclerosis complex (TSC) is a monogenetic, multisystemic disease characterised by the formation of benign tumours that can affect almost all organs, caused by pathogenic variations in *TSC1* or *TSC2*. In this multicentre study from Germany, we investigated the influence of sociodemographic, clinical, and therapeutic factors on quality of life (QoL) among individuals with TSC.

**Methods:**

We assessed sociodemographic and clinical characteristics and QoL among adults with TSC throughout Germany using a validated, three-month, retrospective questionnaire. We examined predictors of health-related QoL (HRQoL) using multiple linear regression analysis and compared the QoL among patients with TSC with QoL among patients with other chronic neurological disorders.

**Results:**

We enrolled 121 adults with TSC (mean age: 31.0 ± 10.5 years; range: 18–61 years, 45.5% [*n* = 55] women). Unemployment, a higher grade of disability, a higher number of organ manifestations, the presence of neuropsychiatric manifestations or active epilepsy, and a higher burden of therapy-related adverse events were associated with worse QoL, as measured by two QoL instruments (EuroQoL-5 dimensions [EQ-5D] and Quality of Life in Epilepsy Patients [QOLIE-31]). Neuropsychiatric and structural nervous system manifestations, the number of affected organs, and therapy-related adverse events were also associated with higher depression, as measured by the Neurological Disorders Depression Inventory for Epilepsy (NDDI-E). In multiple regression analysis, more severe therapy-related adverse events (large effect, *p* < 0.001), active epilepsy (large effect, *p* < 0.001), and neuropsychiatric manifestations (medium effect, *p* = 0.003) were independently associated with worse HRQoL, explaining 65% of the variance (*p* < 0.001). The HRQoL among patients with active TSC-associated epilepsy was worse than that among patients with drug-refractory mesial temporal lobe epilepsy (*p* < 0.001), and the generic QoL among patients with more than three TSC organ manifestations was similar to those of patients with severe migraine and uncontrolled asthma.

**Conclusions:**

Active epilepsy, neuropsychiatric manifestations (such as anxiety and depression), and therapy-related adverse events are important independent predictors of worse quality of life among adults with TSC. Generic quality of life in TSC with several manifestations is similar to uncontrolled severe chronic diseases and significantly negatively correlates with TSC severity.

**Trial registration:**

DRKS, DRKS00016045. Registered 01 March 2019.

## Background

Tuberous sclerosis complex (TSC) is a rare, multisystemic, monogenetic disorder. The incidence of definite or possible TSC is estimated at 1:6760 to 1:13,520 in Germany [1], and TSC prevalence was likely underestimated until recently due to clinical variability [1–4]. TSC is primarily caused by pathogenic variations in the *TSC1* and *TSC2* genes, causing the disinhibition of mechanistic target of rapamycin (mTOR) complex and leading to the dysregulation of cell metabolism, proliferation, and growth and ultimately the formation of benign tumours in multiple organ systems. TSC clinical manifestations vary throughout life, and initially present often in the heart, skin, and brain. Subsequent manifestations can appear in almost every organ, especially the brain, skin, kidneys, eyes, heart, and lungs, resulting in considerable interindividual phenotypic variability among individuals with TSC. Clinical manifestations can range from mild to sufficiently severe to require continuous nursing assistance [2, 5]. Approximately 85–96% of individuals with TSC suffer from structural epilepsy due to the formation of cortical tubers or other malformations [5, 6]. Seizures often manifest within the first 6 months after birth [7]. Other common initial findings include hypomelanotic macules on the skin and cardiac rhabdomyoma, which is often detected prenatally during routine ultrasound examinations and is strongly associated with a TSC diagnosis [5]. Neuropsychiatric problems, including intellectual disability, autism, sleep difficulties, aggression, and anxiety and depression in adults, frequently occur in TSC [5]. Renal angiomyolipoma (AML) and brain subependymal giant cell astrocytoma (SEGA) often present during adolescence, and AML tend to grow during adolescence and adulthood, necessitating life-long surveillance [8]. Pulmonary lymphangioleiomyomatosis almost exclusively affects adult women with TSC [9].

Due to the multifaceted manifestations of TSC, the burden of illness is considerable [10–13]. Studies examining the quality of life (QoL) among those with TSC remain rare. A recent UK study found that QoL was impaired in all individuals with TSC, regardless of the presence of epilepsy and learning disabilities, with the psychosocial domain being the most affected [14]. Another study found worse QoL among those with frequent and more severe seizures [15], supported by a recent Dutch study [16].

However, few studies to date have investigated which aspects of TSC have the strongest effects on QoL. Thus, the present study aimed to provide a comprehensive analysis of QoL among adults with TSC and expand the current knowledge regarding QoL and its predictors by surveying a large, multicentre sample of adults with TSC in Germany.

## Methods

### Patients and survey methods

The present study was designed as a cross-sectional, multicentre survey that enrolled individuals with TSC throughout Germany (Berlin, Bochum, Dresden [Radeberg], Frankfurt, Greifswald, Homburg, Kempten, Lingen [near Münster], Marburg, München, Radeberg [near Dresden], Rostock, Stuttgart, and Wiesbaden) and through the German TSC patient advocacy group (Tuberöse Sklerose Deutschland e.V., Wiesbaden, Germany). Paper questionnaires in German were sent to individuals with TSC between February and July 2019. Participants answered the questionnaire either alone or with caregiver support. In this analysis, we included only adult individuals (≥18 years) who answered at least one of the main patient-reported outcome measures.

After receiving written informed consent from patients or their legal guardians (if applicable), we deemed all individuals with TSC that fulfilled the inclusion criteria as eligible. The diagnostic criteria for TSC were based on the latest recommendations established by the 2012 International TSC Consensus Conference [17]. We identified seven primary TSC manifestation categories such as epilepsy, structural brain defects, psychiatric, heart and circulatory system disorders, kidney and urinary tract disorders, dermatological system manifestations, respiratory system manifestations, and other manifestations [11]. We further identified frequent specific TSC manifestations within each primary category. Seizure and epilepsy syndrome classifications were adapted to the latest definitions established by the International League against Epilepsy [18, 19]. We defined “active epilepsy” as experiencing at least one seizure within the 12 months preceding study participation [20]. This study received ethical approval and was registered with the German Clinical Trials Register (DRKS00016045; Universal Trial Number: U1111–1229-4714).

### Materials

We asked individuals with TSC to complete a retrospective questionnaire based on their experiences during the previous 3 months. The questionnaire was successfully applied in earlier studies [21–25], and we adapted it for use in individuals with TSC. The questionnaire included the following instruments:

1. The EuroQoL–5 dimension–3 level inventory (EQ-5D-3L) measures generic QoL. The EQ-5D comprises a visual analogue scale (VAS, range: 0–100 units), with 100 representing the best and 0 representing the worst health imaginable. The EQ-5D contains 5 three-level Likert questions regarding mobility, self-care, usual activities, pain, and anxiety/depression. A single, continuous summary index is derived by transforming the EQ-5D Likert items with a “value set”. This value set provides prespecified weights for each possible combination of answers according to preferences in a country-specific population. The summary index score ranges from 1 (best imaginable health), 0 (health status equivalent to death) to negative values (health status “worse than death”). We derived summary index values using both the time-trade-off (TTO) and VAS methods, using the “eq5d” package (available from https://cran.r-project.org/web/packages/eq5d/) for R (R Core Team, Vienna, Austria). We report the raw VAS scores alongside the EQ-5D summary index scores.
2. The Quality of Life in Epilepsy Inventory–31 items (QOLIE-31) measures health-related QoL (HRQoL) [26], which has been deemed appropriate for individuals with TSC [27]. The QOLIE-31 features 30 items across 7 dimensions (overall QoL, seizure worry, emotional well-being, energy/fatigue, cognitive function, medication effects, and social functioning) and a separate VAS analogous to the VAS in the EQ-5D. We calculated QOLIE-31 scores based on the manual (available from [28]). In brief, items in each of the 7 dimensions were first combined into subscale scores. A continuous overall score was obtained as a summary of the weighted subscores. The QOLIE-31 overall score ranges from 0 to 100, with 100 representing the best overall disease-related health and 0 representing the worst health. The VAS is not included in the overall QOLIE-31 score but is reported separately.
3. The Neurological Disorders Depression Inventory for Epilepsy (NDDI-E) [29] is an externally validated instrument for assessing depression, developed for a cohort of epilepsy patients. The NDDI-E features 6 four-level Likert items regarding the frequency of common depressive symptoms, such as feeling guilty, frustrated, or weary of life. An aggregate value of ≥14 points (range: 6–24 points) is indicative of depressive mood in the validated German version [27].
4. The Epilepsy Stigma Scale measures disease-related stigma [28], using three questions (‘I feel that some people are uncomfortable with me’, ‘I feel some people treat me like an inferior person,’ and ‘I feel some people would prefer to avoid me’). An affirmative answer to any question indicates stigma, with an aggregate value of 3 suggesting a severe stigma [27]. We dichotomised the Epilepsy Stigma Scale into ‘severe stigma’ and ‘no or not severe stigma’, according to Baker et al. [27].
5. We measured therapy-related adverse events with the Liverpool Adverse Events Profile (LAEP) inventory, which features 20 four-level Likert items using summarised item values (range 20–80). Based on previous cohorts, a cutoff score of 35 points was used to indicate relevant therapy-related adverse events [30].
6. We quantitatively assessed the TSC-related burden of illness using 20 four-level Likert items, each measuring the severity of burden from a possible TSC organ manifestation, and 33 additional questions regarding disease characteristics (e.g. genetics, affected organ systems, seizures, medications, and additional symptoms), healthcare resource use, and social situations.

In the German social system, individuals with disabilities who are entitled to certain monetary and social compensations are assigned a ‘grade of disability’, which quantifies the disability type and severity and determines compensation. The grade of disability is classified by an independent medical professional and ranges from 20 to 100, in steps of 10.

### Statistical analysis

Descriptive analyses were conducted for sociodemographic and clinical characteristics. The following variables were analysed as potential QoL factors: age (median split for univariate analysis), sex (male/female), employment (yes/no), grade of disability (≤80/> 80), mutation type (*TSC1*/*TSC2*), active epilepsy (yes/no), structural central nervous system manifestation (yes/no), SEGA (yes/no), neuropsychiatric manifestation (yes/no), renal AML (yes/no), lymphangioleiomyomatosis (yes/no), skin manifestation (yes/no), number of manifestations (quartile split for univariate analysis), anti-seizure medication polytherapy (< 2 /≥2 medications), use of everolimus (yes/no), and LAEP score (< 35/≥35). Generic QoL (EQ-5D), HRQoL (QOLIE-31), and mood (NDDI-E) results were compared relative to these potential factors using the Kruskal–Wallis-test, Chi-square test, and independent-samples *t*-test. The association between (HR) QoL and burden of illness and between HRQoL and LAEP score, number of organ manifestations, and grade of disability were assessed using correlation analysis (Pearson’s correlation coefficient).

To evaluate potential predictors of HRQoL (dependent variable: QOLIE-31 overall score), we performed multiple linear regression analysis. All predictors defined as significant in the group comparisons that broadly overlapped with known predictors from the literature were included, and a non-hierarchical forced entry method was applied. We calculated effect sizes for significant individual predictors in the regression model based on the squares of the semi-partial predictor correlations [*r*^*2*^_*part*_ / (1 − *r*^*2*^_*part*_)], with cutoffs of *f*^*2*^ ≥ 0.02 for small, *f*^*2*^ ≥ 0.15 for medium, and *f*^*2*^ ≥ 0.35 for large effect sizes, based on Cohen’s suggestions [31]. According to power estimates by Miles and Shevlin [32], our sample size allowed for the detection of medium and large effect sizes in a five-predictor model. We assessed the multicollinearity of all predictors and used tolerance as a measure of collinearity, with a threshold of 0.25, below which collinearity was assumed. To assess predictors of severe disease-related stigma (dependent variable: Epilepsy Stigma Scale = 3 vs fewer points), we used binomial logistic regression.

A *p*-value < 0.05 (two-sided) was considered significant. We adjusted for multiple comparisons using the false-discovery rate method established by Benjamini and Hochberg [33], with a threshold for the false-discovery rate of 0.05. All *p*-values provided in the results section are significant after correction for multiple comparisons. In the tables, all *p*-values that remain significant after correction for multiple comparisons are marked with (†) and bold type. Statistical analysis was performed using IBM SPSS Statistics, version 26 (IBM Corp., Armonk, NY, USA) and R, version 3.6.2 (R Core Team, Vienna, Austria).

## Results

### Demographic and clinical characteristics

In total, 121 individuals were included in this analysis, with an average age of 31.0 years (standard deviation [*SD*] = 10.5 years, range: 18–61 years), and 45.5% (*n* = 55) were women. The median (interquartile range [IQR]) age at first TSC manifestation was during the first year of life (IQR: 0–3 years), and the median age of formal TSC diagnosis of TSC was 2.5 years (IQR: 0–14.3 years). Individuals had a median of 5 TSC manifestations (range: 1–8). The most commonly affected organ systems were the skin (95.9%, *n* = 116) and brain, including epilepsy/seizures (76.9%, *n* = 93) and structural brain manifestations (71.1%, *n* = 86). The most common specific manifestations were facial angiofibroma (83.5%, *n* = 101), seizures (76.9%, *n* = 93), renal AML (57.9%, *n* = 70), hypomelanotic macules (56.2%, *n* = 68), and cortical tubers (52.9%, *n* = 64). Neuropsychiatric manifestations were reported by 60 (49.6%) respondents. Among individuals with seizures, 53.8% (*n* = 50) had active epilepsy, indicating that they experienced at least one seizure during the 12 months preceding the date of study participation. Almost half of the study population (46.3%, *n* = 56) reported ≥12 years of formal education. Of the 83 individuals that disclosed employment status, 41.0% (*n* = 34) were currently unemployed. Additional clinical and demographic information is provided in Table 1.

**Table 1:** Sociodemographic and clinical characteristics of participants (*n* = 121)

		All patients, ***n*** = 121
Age, years	Mean ± *SD*	31.0 ± 10.5
	Range	18–61
	Median	29.0
	IQR	22–37
Sex, *n* (%)	Female	55 (45.5)
	Male	66 (55.5)
Age at first manifestation of TSC, years	Mean ± *SD*	3.8 ± 8.1
	Range	0–41
	Median	0
	IQR	0–3
Age at TSC diagnosis, years	Mean ± *SD*	8.9 ± 13.0
	Range	0–53
	Median	2.5
	IQR	0–14.3
First TSC symptom/sign, *n* (%)	Epilepsy/seizures	65 (53.7)
	Skin manifestation	24 (19.8)
	Cardiac rhabdomyoma	7 (5.8)
	Renal manifestation	4 (3.3)
	Eye manifestation	3 (2.5)
	Cardiac arrhythmia	3 (2.5)
	Other	12 (9.9)
Genetic testing, *n* (%)	Yes, *TSC1*	25 (20.7)
	Yes, *TSC2*	31 (25.6)
	Yes, *TSC2*/*PKD1* contiguous gene	1 (0.8)
	Yes, but no mutation identified (NMI)	16 (13.2)
	Yes, mutation not specified	10 (8.3)
	No genetic testing	30 (24.8)
	Unknown	8 (6.6)
Clinical manifestations, *n* (%)	Skin	116 (95.9)
	Angiofibroma	101 (83.5)
	Hypomelanotic macules	68 (56.2)
	Shagreen patches	59 (48.8)
	Ungual fibromas	13 (10.7)
	Skin tags	4 (3.3)
	Café au lait-spots	4 (3.3)
	Epilepsy/seizures	93 (76.9)
	Active epilepsy	50 (41.3)
	Brain structural	86 (71.1)
	Tuber	64 (52.9)
	SEGA	51 (42.1)
	Hydrocephalus	3 (2.5)
	Kidney	85 (70.2)
	AML	70 (57.9)
	Heart	62 (51.2)
	Cardiac rhabdomyoma	29 (24.0)
	Neuropsychiatric	60 (49.6)
	Lymphangioleiomyomatosis	12 (9.9)
	Other	50 (41.3)
Number of manifestations	Median	5
	Range	1–8
Highest school graduation certificate, *n* (%)	High school (‘Abitur/Fachabitur’)	29 (24.0)
	Intermediate school (‘Realschulabschluss/mittlere Reife’)	27 (22.3)
	Secondary modern school (‘Hauptschulabschluss’)	10 (8.3)
	Still in school	6 (5.0)
	No certificate	29 (24.0)
	Other	19 (15.7)
Current occupation, *n* (%)	Employee full-time	35 (28.9)
	Unemployment or unable to work due to TSC	29 (23.4)
	Employee part-time	12 (9.9)
	Unemployment or unable to work due to other illness	5 (4.1)
	Self-employed	2 (1.7)

*AML* angiomyolipoma, *IQR* interquartile range, *PKD* polycystic kidney disease, *SEGA* subependymal giant cell astrocytoma, *SD* standard deviation, *TSC* tuberous sclerosis complex

### Generic and health-related quality of life, mood, and stigma

#### EQ-5D

For the entire study population, the EQ-5D TTO summary index score was 0.818 (*SD* = 0.262, range: − 0.140 to 1, Table 2). Generic QoL was lower among unemployed (mean = 0.661, *SD* = 0.341) than employed individuals (mean = 0.920, *SD* = 0.115; *p* < 0.001), and lower among those with a disability grade above 80 (mean = 0.681, *SD* = 0.318) compared with those with lower grades (mean = 0.916, *SD* = 0.101; *p* < 0.001). QoL did not differ between sex and age categories. QoL was worse among those with active epilepsy (mean = 0.729, *SD* = 0.305) than in those who were seizure-free over the past 12 months (mean = 0.883, *SD* = 0.205; *p* = 0.001). Those with neuropsychiatric manifestations had significantly worse QoL than those without (mean = 0.693, *SD* = 0.314 vs mean = 0.938, *SD* = 0.107; *p* < 0.001). Those with higher numbers of manifestations reported worse QoL than those less affected by TSC (1–3 manifestations: mean = 0.931, *SD* = 0.159; 4–6 manifestations mean = 0.805, *SD* = 0.264; 7–8 manifestations: mean = 0.662, *SD* = 0.330; *p* = 0.001; Table 2). QoL did not differ significantly between those with *TSC1* and *TSC2* variations. The presence of renal AML, skin manifestations, structural brain manifestations (specifically SEGA), and lung manifestations (lymphangioleiomyomatosis) were not associated with worse generic QoL. Only 12 individuals reported the presence of lymphangioleiomyomatosis. Anti-seizure medication polytherapy was not associated with a worse generic QoL, nor was the use of everolimus (in the TTO index). However, those with a LAEP score ≥ 35 points, indicating moderate-to-severe therapy-related adverse events, had significantly worse QoL than those with milder therapy-related adverse events (mean = 0.757, *SD* = 0.287 vs mean = 0.960, *SD* = 0.093; *p* < 0.001). The EQ-5D VAS summary index demonstrated significant differences for the same variables as the TTO summary index, plus everolimus use. Individuals who used everolimus reported worse QoL than those without everolimus use in the VAS (*p* = 0.007) but not the TTO index (*p* = 0.141). Using individual EQ-5D subscales, the highest frequency of individuals reporting any degree of problems was identified in the usual care (47.5%) and anxiety/depression domains (40.5%), followed by self-care (37.4%), pain (27.0%), and mobility (12.4%).

**Table 2:** Comparisons of generic quality of life among individuals with TSC, as measured by the EQ-5D (German version) questionnaire, according to several potential predictors and assessed by the Kruskal–Wallis and Chi-square tests

Predictor	Measure	***N***	Category	Mean	± ***SD***	95% CI	***p***-value*	
**Sociodemographic aspects**								
Sex	VAS	64	Male	67.9	19.7	63.0–72.9	0.768	
		54	Female	66.7	21.2	60.9–72.5		
	Index (TTO)	64	Male	0.796	0.267	0.730–0.863	0.320	
		54	Female	0.843	0.257	0.773–0.914		
Age	VAS	54	18–28 y	72.0	20.4	66.4–77.6	0.020	
		62	29–61 y	68.2	19.6	58.3–68.2		
	Index (TTO)	54	18–28 y	0.834	0.238	0.769–0.898	0.309	
		62	29–61 y	0.800	0.286	0.727–0.873		
Employment	VAS	49	Yes	70.8	18.2	65.6–76.0	0.017	
		33	No	58.7	21.7	51.0–66.4		
	Index (TTO)	49	Yes	0.920	0.115	0.887–0.953	**< 0.001†**	
		33	No	0.661	0.341	0.540–0.782		
Grade of disability	VAS	36	0–80	72.0	15.9	66.6–77.4	**0.001†**	
		56	90–100	58.6	20.3	53.1–64.0		
	Index (TTO)	36	0–80	0.916	0.101	0.882–0.950	**< 0.001†**	
		56	90–100	0.681	0.318	0.596–0.766		
**Clinical aspects**								
Mutation type	VAS	24	*TSC1*	72.8	18.5	65.0–80.6	0.198	
		30	*TSC2*	65.0	24.2	56.0–74.1		
	Index (TTO)	24	*TSC1*	0.803	0.288	0.682–0.924	0.662	
		30	*TSC2*	0.793	0.296	0.682–0.904		
Active epilepsy	VAS	50	Yes	60.0	20.1	54.3–65.7	**0.001†**	
		71	Sz freedom > 12 m	72.3	18.9	68.2–77.4		
	Index (TTO)	50	Yes	0.729	0.305	0.642–0.816	**0.001†**	
		71	Sz freedom > 12 m	0.883	0.205	0.834–0.939		
Structural brain	VAS	83	Yes	65.4	20.3	61.0–69.9	0.116	
		35	No	72.0	20.0	65.1–78.8		
	Index (TTO)	83	Yes	0.793	0.281	0.732–0.855	0.123	
		35	No	0.876	0.206	0.805–0.947		
SEGA	VAS	50	Yes	62.2	20.5	56.3–68.0	0.017	
		68	No	71.2	19.5	66.5–75.9		
	Index (TTO)	50	Yes	0.802	0.268	0.725–0.877	0.597	
		68	No	0.830	0.260	0.767–0.893		
Neuropsychiatric	VAS	60	Yes	59.1	21.4	53.5–64.7	**< 0.001†**	
		61	No	75.4	15.7	71.3–75.4		
	Index (TTO)	60	Yes	0.693	0.314	0.610–0.776	**< 0.001†**	
		61	No	0.938	0.107	0.911–0.966		
AML	VAS	69	Yes	65.9	21.2	60.8–71.0	0.317	
		49	No	69.4	19.2	63.9–74.9		
	Index (TTO)	70	Yes	0.805	0.276	0.739–0.872	0.979	
		51	No	0.835	0.244	0.765–0.906		
Lymphangioleio-myomatosis	VAS	12	Yes	67.5	12.7	59.4–75.6	0.844	
		106	No	67.4	21.1	63.3–71.4		
	Index (TTO)	12	Yes	0.819	0.320	0.615–1.023	0.839	
		106	No	0.818	0.257	0.768–0.867		
Skin	VAS	114	Yes	67.1	20.5	63.3–70.9	0.469	
		4	No	75.0	13.5	63.3–96.6		
	Index (TTO)	116	Yes	0.815	0.267	0.767–0.865	0.504	
		5	No	0.890	0.087	0.753–1.028		
Number of affected organs	VAS	30	1–3	80.9	12.3	76.3–85.5	ref.	**< 0.001†**
		72	4–6	64.5	20.3	59.7–69.3	**0.005**	
		16	7–8	55.1	20.3	44.2–65.9	**0.048**	
	Index (TTO)	30	1–3	0.931	0.159	0.872–0.990	ref.	**0.001†**
		72	4–6	0.805	0.264	0.743–0.867	**0.037**	
		16	7–8	0.662	0.330	0.487–0.838	**0.009**	
**Therapeutic aspects**								
Anti-seizure medication polytherapy^a^	VAS	53	Yes	62.3	20.2	61.7–74.9	0.223	
		37	No	68.3	19.8	57.3–68.4		
	Index (TTO)	53	Yes	0.759	0.282	0.682–0.837	0.081	
		40	No	0.851	0.250	0.767–0.934		
Everolimus	VAS	50	Yes	61.6	20.3	55.8–67.4	**0.007†**	
		68	No	71.6	19.4	66.9–76.3		
	Index (TTO)	50	Yes	0.772	0.286	0.691–0.854	0.141	
		68	No	0.851	0.241	0.793–0.910		
LAEP	VAS	34	< 35	62.6	19.7	58.3–66.9	**< 0.001†**	
		83	≥35	78.4	17.5	72.3–84.5		
	Index (TTO)	34	< 35	0.757	0.287	0.695–0.820	**< 0.001†**	
		83	≥35	0.960	0.093	0.928–0.993		

*AML* angiomyolipoma, *CI* confidence interval, *EQ-5D* EuroQoL-5 dimensions, *TTO* time-trade-off method, *VAS* visual analogue scale, *LAEP* Liverpool Adverse Events Profile, *m* months, *QOLIE-31* Quality of Life in Epilepsy Inventory-31 items, *ref*. reference category, *SD* standard deviation, *SEGA* subependymal giant cell astrocytoma, *sz* seizure, *TSC* tuberous sclerosis complex, *y* years

^a^Includes only individuals with TSC-associated epilepsy/seizures

*Comparisons corrected for multiple testing using the Benjamini–Hochberg false-discovery rate method, † and bold type denotes a *q*-value of < 0.05 (false discovery rate)

Visual analysis of a histogram displaying both the TTO- and VAS-transformed EQ-5D summary index scores for the whole study population identified a highly right-skewed distribution. Further comparison of the QOLIE-31 overall scores and the EQ-5D summary indices demonstrated a large share of maximum EQ-5D summary index values among individuals with QOLIE-31 overall scores down to about 62.5, indicating a substantial ceiling effect for the EQ-5D instrument in our cohort.

#### QOLIE-31

For the overall study population, the QOLIE-31 overall score was 63.5 (*SD* = 19.7, range: 12.15–100; Table 3). The sociodemographic, clinical, and therapy-related variables associated with a worse HRQoL were generally identical to those identified by the EQ-5D summary index score analysis. The QOLIE-31 overall score was lower among unemployed than employed individuals (mean = 51.5, *SD* = 16.5 vs mean = 71.7, *SD* = 19.9; *p* < 0.001) and among those with a disability grade above 80 compared with those with lower grades (mean = 54.2, *SD* = 16.5 vs mean = 66.1, *SD* = 16.5; *p* < 0.001). Scores were lower in those with active epilepsy than in 12-month seizure-free individuals (mean = 52.1, *SD* = 17.7 vs mean = 71.7, *SD* = 16.7; *p* < 0.001) and in those with neuropsychiatric manifestations compared with those without (mean = 53.2, *SD* = 18.0 vs. mean = 73.8, *SD* = 15.4; *p* < 0.001). Those with 1–3 TSC manifestations had better HRQoL than those with 4–6 or 7–8 manifestations (mean = 71.7, *SD* = 18.4 vs mean = 62.8, *SD* = 18.9 vs mean = 52.9, *SD* = 20.0; *p* = 0.007; Table 3). Those with structural brain manifestation (not specifically SEGA) had worse HRQoL than those without (mean = 60.5, *SD* = 20.1 vs mean = 71.5, *SD* = 16.3, *p* = 0.007) but only for the overall score, not the VAS (*p* = 0.232). No other clinical aspects significantly influenced HRQoL. Those with a LAEP of ≥35 points had significantly worse HRQoL than those more mildly affected (mean = 56.0, *SD* = 17.2 vs mean = 80.5, *SD* = 12.7; *p* < 0.001).

**Table 3:** Comparisons of health-related quality of life among individuals with TSC, as measured by the QOLIE-31 (German version) questionnaire, according to several potential predictors and assessed using the Kruskal–Wallis and Chi-square tests

Predictor	Measure	***N***	Category	Mean	± ***SD***	95% CI	***p***-value*	
**Sociodemographic aspects**								
Sex	VAS	65	Male	64.2	19.9	59.2–69.1	0.790	
		55	Female	63.6	22.1	57.6–69.5		
	Overall score	65	Male	62.2	20.2	57.5–67.2	0.277	
		55	Female	65.5	19.0	60.3–70.6		
Age	VAS	55	18–28 y	67.1	20.3	61.6–72.6	0.136	
		63	29–61 y	60.9	21.0	55.6–66.2		
	Overall score	55	18–28 y	64.4	20.6	58.9–70.0	0.666	
		53	29–61 y	62.7	19.0	57.9–67.5		
Employment	VAS	49	Yes	70.6	19.7	64.9–76.2	**0.001†**	
		34	No	54.7	20.8	46.4–60.9		
	Overall score	49	Yes	71.7	19.9	66.9–76.4	**< 0.001†**	
		34	No	51.5	16.5	44.5–58.4		
Grade of disability	VAS	38	0–80	65.0	17.4	59.2–70.7	**0.008†**	
		56	90–100	54.3	18.1	49.4–59.1		
	Overall score	38	0–80	66.1	16.5	60.7–71.5	**< 0.001†**	
		56	90–100	54.2	16.5	49.7–58.6		
**Clinical aspects**								
Mutation type	VAS	25	*TSC1*	70.3	18.0	62.9–77.8	0.099	
		31	*TSC2*	61.0	23.5	52.3–69.6		
	Overall score	25	*TSC1*	68.8	17.3	61.6–75.9	0.424	
		31	*TSC2*	63.0	21.4	55.2–70.9		
Active epilepsy	VAS	49	Yes	56.7	19.4	51.2–62.3	**0.003†**	
		71	No	68.8	20.5	64.0–73.7		
	Overall score	49	Yes	52.1	17.7	47.0–57.2	**< 0.001†**	
		71	No	71.7	16.7	67.8–75.7		
Structural brain	VAS	85	Yes	62.3	20.9	57.8–66.8	0.232	
		35	No	67.7	20.5	60.6–74.7		
	Overall score	85	Yes	60.5	20.1	56.2–64.8	**0.007†**	
		35	No	71.5	16.3	65.9–77.1		
SEGA	VAS	50	Yes	62.0	22.1	55.7–68.3	0.458	
		70	No	65.2	20.0	60.5–70.0		
	Overall score	50	Yes	59.4	20.1	53.7–65.2	0.041	
		70	No	66.7	18.9	62.2–71.2		
Neuropsychiatric	VAS	59	Yes	55.1	20.4	49.8–60.5	**< 0.001†**	
		61	No	72.3	17.6	67.8–76.9		
	Overall score	59	Yes	53.2	18.0	48.5–57.9	**< 0.001†**	
		61	No	73.8	15.4	69.9–77.8		
AML	VAS	70	Yes	61.5	22.3	56.2–66.9	0.146	
		50	No	67.2	18.4	61.9–72.4		
	Overall score	70	Yes	63.6	20.8	58.6–68.5	0.819	
		50	No	63.9	18.2	58.7–69.0		
Lymphangioleiomyomatosis	VAS	12	Yes	63.3	14.4	54.2–72.5	0.829	
		108	No	63.9	21.5	59.8–68.1		
	Overall score	12	Yes	70.2	14.9	60.8–79.7	0.256	
		108	No	63.0	20.0	59.2–66.8		
Skin	VAS	115	Yes	63.8	20.9	59.9–67.7	0.822	
		5	No	66.0	20.7	40.3–91.8		
	Overall score	115	Yes	63.7	19.6	60.1–67.3	0.855	
		5	No	63.3	22.9	34.8–91.8		
Number of affected organs	VAS	31	1–3	76.7	16.7	70.6–82.8	Ref	**< 0.001†**
		72	4–6	62.2	19.2	57.7–66.7	**0.001**	
		17	7–8	47.7	21.6	36.6–58.8	**< 0.001**	
	Overall score	31	1–3	71.7	18.4	65.0–78.5	Ref	**0.007†**
		72	4–6	62.8	18.9	58.4–67.2	**0.026**	
		17	7–8	52.9	20.0	42.6–63.2	**0.002**	
**Therapeutic aspects**								
Anti-seizure medication polytherapy^a^	VAS	52	Yes	58.1	19.0	52.8–64.4	0.178	
		31	No	64.8	20.1	57.4–72.2		
	Overall score	52	Yes	54.9	17.8	49.9–59.8	0.032	
		31	No	63.5	18.6	56.6–70.3		
Everolimus	VAS	51	Yes	59.3	22.0	53.1–65.5	0.063	
		69	No	67.3	19.4	62.6–71.9		
	Overall score	51	Yes	60.5	20.0	54.9–66.1	0.212	
		69	No	66.1	19.1	61.5–70.7		
LAEP	VAS	36	< 35	76.7	18.0	70.6–82.8	**< 0.001†**	
		83	≥35	58.0	19.3	53.8–62.2		
	Overall score	36	< 35	80.5	12.7	76.2–84.7	**< 0.001†**	
		83	≥35	56.0	17.2	52.3–59.8		

*AML* angiomyolipoma, *CI* confidence interval, *LAEP* Liverpool Adverse Events Profile, *QOLIE-31* Quality of Life in Epilepsy Inventory-31 items, *ref*. reference category, *SD* standard deviation, *SEGA* subependymal giant cell astrocytoma, *TSC* tuberous sclerosis complex, *VAS* visual analogue scale, *y* years

^a^Includes only individuals with TSC-associated epilepsy/seizures

*Comparisons corrected for multiple testing using the Benjamini–Hochberg false-discovery rate method, † and bold type denotes a *q*-value of < 0.05 (false-discovery rate)

**Fig. 1:**
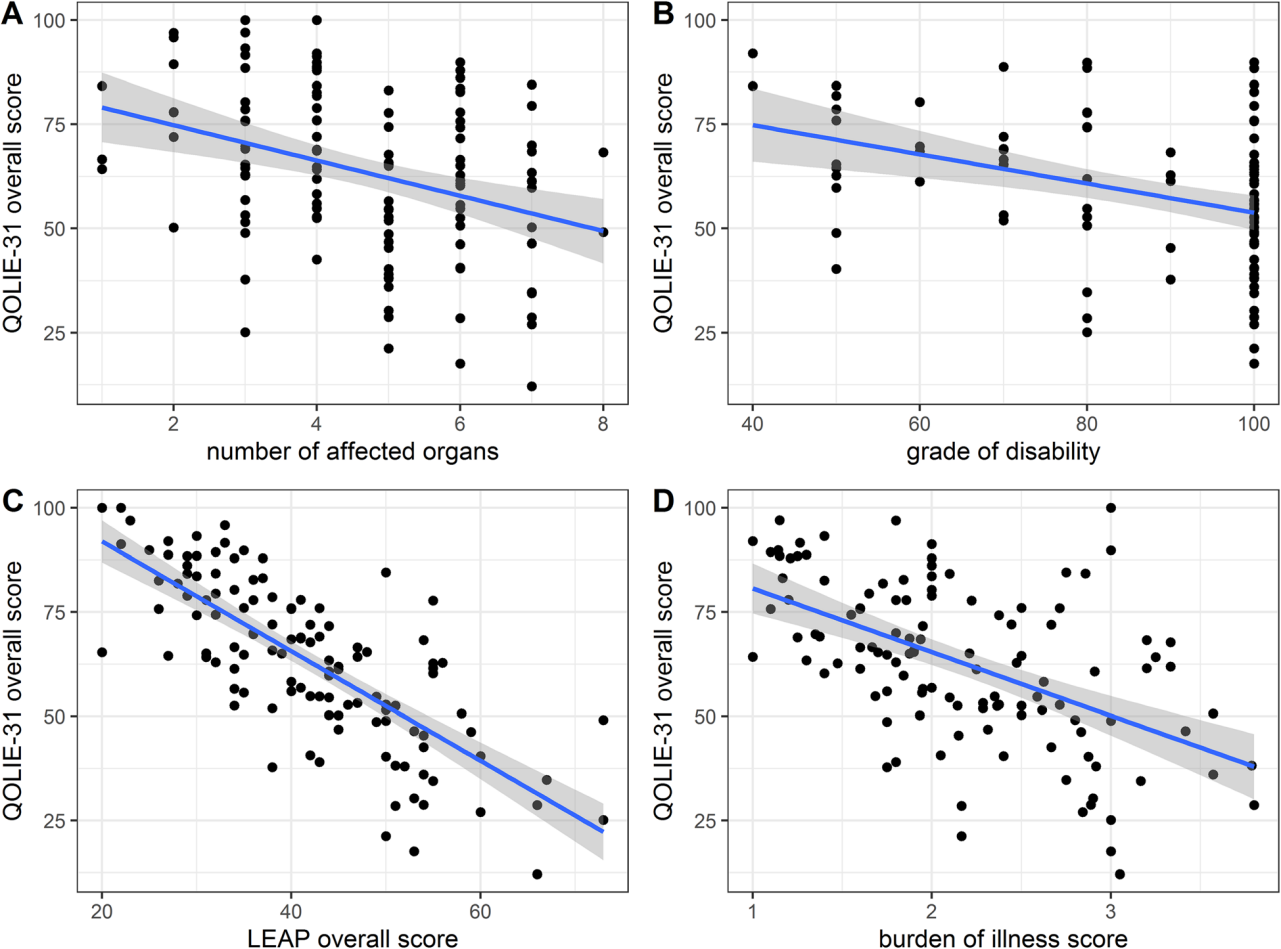
Correlation between health-related quality of life and other factors in TSC. Health-related quality of life (HRQoL) in adult individuals with tuberous sclerosis complex (TSC), as assessed by the Quality of Life in Epilepsy Inventory-31 items (QOLIE-31) overall score was significantly correlated with **A**) the number of affected organ systems, **B**) the grade of disability, and **C**) the overall Liverpool Adverse Events Profile (LAEP) score, which measures therapy-related adverse events. **D**: HRQoL in adults with TSC was also significantly correlated with the perceived burden of illness, which was calculated as an index of 20 possible TSC-associated problems (possible range of 0–4 for each item)

The number of manifestations (*r* = − 0.35, 95% confidence interval (CI): − 0.49 to − 0.18, *p* < 0.001), grade of disability (*r* = − 0.38, 95% CI: − 0.54 to − 0.19, *p* < 0.001) and LAEP score (*r* = − 0.76, 95% CI: − 0.83 t o − 0.67, *p* < 0.001) were each significantly correlated with the QOLIE-31 overall score (Fig. 1).

#### NDDI-e

The mean NDDI-E value in our cohort was 12.4 (median = 12, range: 6–23), only slightly below the cutoff of 14 points for probable depression. Several variables were associated with worse NDDI-E values (the presence of structural brain or neuropsychiatric manifestations, a higher number of affected organs, and LAEP ≥35 points). However, only the subgroup with the most TSC manifestations had a mean NDDI-E greater than 14 points. The median reached 14 points in those with at least six TSC manifestations, indicating that at least 50% of these severely affected individuals are likely to show depression symptoms (Table 4).

**Table 4:** Comparisons of mood among individuals with TSC, as measured by the NDDI-E (German version) questionnaire, according to several potential predictors and assessed using Kruskal–Wallis and Chi-square tests

Predictor	***N***	Category	Mean	± ***SD***	95% CI	***p***-value*
**Sociodemographic aspects**						
Sex	66	Male	12.32	4.0	11.3–13.3	0.767
	55	Female	12.58	4.2	11.5–13.7	
Age	56	18–28 y	11.8	4.0	10.8–12.9	0.192
	63	29–61 y	13.0	4.2	11.9–14.0	
Employment	49	Yes	11.8	3.9	10.7–12.9	0.056
	34	No	13.8	4.6	12.2–15.9	
Grade of disability	38	0–80	12.5	4.1	11.2–13.9	0.5327
	57	90–100	13.2	3.6	12.2–14.2	
**Clinical aspects**						
Mutation type	25	*TSC1*	12.4	4.5	10.6–14.3	0.791
	31	*TSC2*	12.3	3.6	10.9–13.6	
Active epilepsy	50	Yes	13.2	3.8	12.2–14.3	0.074
	71	No	11.9	4.2	10.9–12.9	
Structural brain	86	Yes	13.1	4.1	12.3–14.0	**0.004†**
	35	No	10.7	3.5	9.5–11.9	
SEGA	51	Yes	13.1	4.2	12.0–14.3	0.046
	70	No	11.9	4.0	11.0–12.9	
Neuropsychiatric	60	Yes	13.7	4.0	12.6–14.7	**0.001†**
	61	No	11.3	3.8	10.3–12.2	
AML	70	Yes	12.3	4.3	11.3–13.4	0.866
	51	No	12.6	3.8	11.5–13.7	
Lymphangioleiomyomatosis	12	Yes	12.2	4.1	9.5–14.8	0.878
	109	No	12.5	4.1	11.7–13.3	
Skin	116	Yes	12.3	4.0	11.6–13.1	0.281
	5	No	14.6	5.9	7.3–21.9	
Number of affected organs	31	1–3	11.4	4.1	9.8–12.9	**0.006†**
	73	4–6	12.3	4.0	11.4–13.3	
	17	7–8	14.8	3.6	13.0–16.7	
**Therapeutic aspects**						
Anti-seizure medication polytherapy^a^	53	Yes	12.8	3.5	11.9–13.8	0.487
	31	No	13.6	4.1	12.0–15.1	
Everolimus	51	Yes	12.7	4.1	11.6–13.9	0.351
	70	No	12.2	4.1	11.3–13.2	
LAEP	36	< 35	10.0	3.7	8.7–11.2	**< 0.001†**
	84	≥35	13.6	3.7	12.8–14.4	

*AML* angiomyolipoma, *CI* confidence interval, *LAEP* Liverpool Adverse Events Profile, *NDDI-E* Neurological Disorders Depression Inventory for Epilepsy, *ref*. reference category, *SD* standard deviation, *SEGA* subependymal giant cell astrocytoma, *TSC* tuberous sclerosis complex, *VAS* visual analogue scale, *y* years

^a^Includes only individuals with TSC-associated epilepsy/seizures

*comparisons corrected for multiple testing using the Benjamin Hochberg false-discovery rate method, † and bold type denotes a *q*-value of < 0.05 (false-discovery rate)

#### Epilepsy stigma scale

Severe stigma (Epilepsy Stigma Scale = 3 points) was associated with neuropsychiatric manifestations (*p* = 0.004), epilepsy (*p* = 0.011, but not active epilepsy [*p* = 0.145]), structural brain manifestations (*p* = 0.029), SEGA (*p* = 0.029), and worse LAEP scores (*p* < 0.001). In binomial logistic regression, only the LAEP score independently predicted severe stigma, with each point increase in LAEP score increasing the odds of perceiving a severe stigma by 7% (estimate: 0.067, standard error: 0.02, *p* = 0.001).

### Association between quality of life and burden of illness

Both generic QoL and HRQoL were highly correlated with a 20-item burden of illness index (item sum/number of noted items). The EQ-5D correlated negatively with the burden of illness index (*r* = − 0.53, 95% CI: − 0.65 to − 0.39, *p* < 0.001), indicating that higher disease burden was associated with worse generic QoL. The HRQoL was similarly correlated with increasing burden of illness (*r* = − 0.53, 95% CI: − 0.65 to − 0.39, *p* < 0.001; Fig. 1).

### Comparison of (health-related) quality of life between disorders

**Fig. 2:**
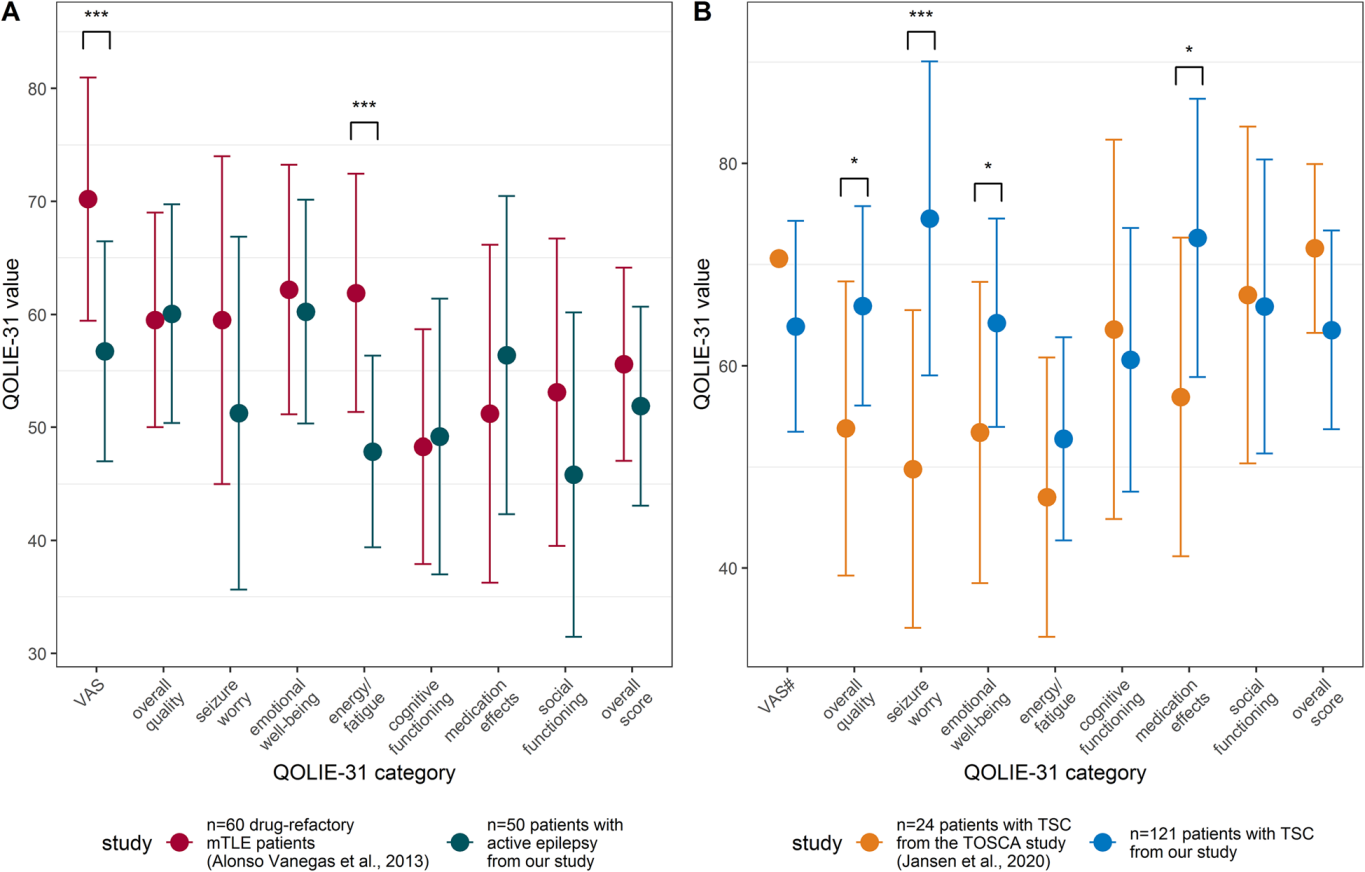
Health-related quality of life in our TSC cohort compared with other cohorts with epilepsy. **A:** Comparison of health-related quality of life (HRQoL), as assessed by the Quality of Life in Epilepsy Inventory-31 items (QOLIE-31) between a study on patients with drug-refractory mesial temporal lobe epilepsy and individuals from our tuberous sclerosis complex (TSC) cohort with active epilepsy (defined as experiencing seizures within the last 12 months). The TSC patients in our cohort demonstrated a lower overall health status compared with temporal lobe epilepsy patients, as measured by the visual analogue scale (VAS) component of the QOLIE-31. Fatigue was higher among TSC patients than temporal lobe epilepsy patients. **B:** Comparison of patients with TSC, both with and without epilepsy, between the TOSCA registry and our overall cohort. The QOLIE-31 overall quality of life subscore was significantly worse in the TOSCA cohort, seizure worry was higher, emotional well-being was worse, and medication effects were higher among the TOSCA cohort compared with our cohort, with all other categories demonstrating similar values. #Standard deviation for the VAS scale of the questionnaire was not reported by Jansen et al. Independent samples *t*-tests, * = *p* < 0.05, ** = *p <* 0.01, *** = *p* < 0.001

The HRQoL-VAS was lower among our active epilepsy cohort than among a cohort of 60 patients with drug-refractory epilepsy [34] (Fig. 2a; *p* < 0.001, *t*-test). Normative values exist for the QOLIE-31 subscales, allowing external comparisons based on *T*-scores (*T* = 50 represents the average value for each subscale) [35]. We found a mean “seizure worry” score of 67.0 (*SD* = 31.7) among our 93 individuals with TSC-associated epilepsy, indicating a slightly lower seizure worry than average (*T* = 53) [36] and compared with 24 patients of a large registry of individuals with TSC (TuberOus SClerosis registry to increase disease Awareness [TOSCA]) [16] (Fig. 2b, *p* < 0.001, *t*-test). The overall QoL subscale value in our TSC-epilepsy cohort was slightly below average (mean = 62.9, *SD* = 18.8, *T* = 48) but better than that in the comparator registry (*p* = 0.0128, *t*-test). Emotional well-being was slightly below average in our cohort (mean = 61.9, *SD* = 20.0, *T* = 47) but better than that in the TOSCA registry (*p* = 0.0321, *t*-test). Fatigue (mean = 50.7, *SD* = 18.4), cognitive functioning (mean = 54.7, *SD* = 24.7), and social functioning (mean = 58.8, *SD* = 28.5) in our patients were all slightly below average for an epilepsy cohort (*T* = 47); however, fatigue was higher in our active epilepsy subgroup than in drug-refractory medial temporal lobe epilepsy patients [34] (Fig. 2a, *p* < 0.001, *t*-test). Medication-associated adverse effects were average compared with an epilepsy cohort (mean = 65.7, *SD* = 24.2, *T* = 53) but lower than in the comparator registry (*p* = 0.0138, *t*-test). Figure 2 shows additional non-significant differences between our cohort and the drug-refractory temporal lobe epilepsy and TOSCA cohorts.

**Fig. 3:**
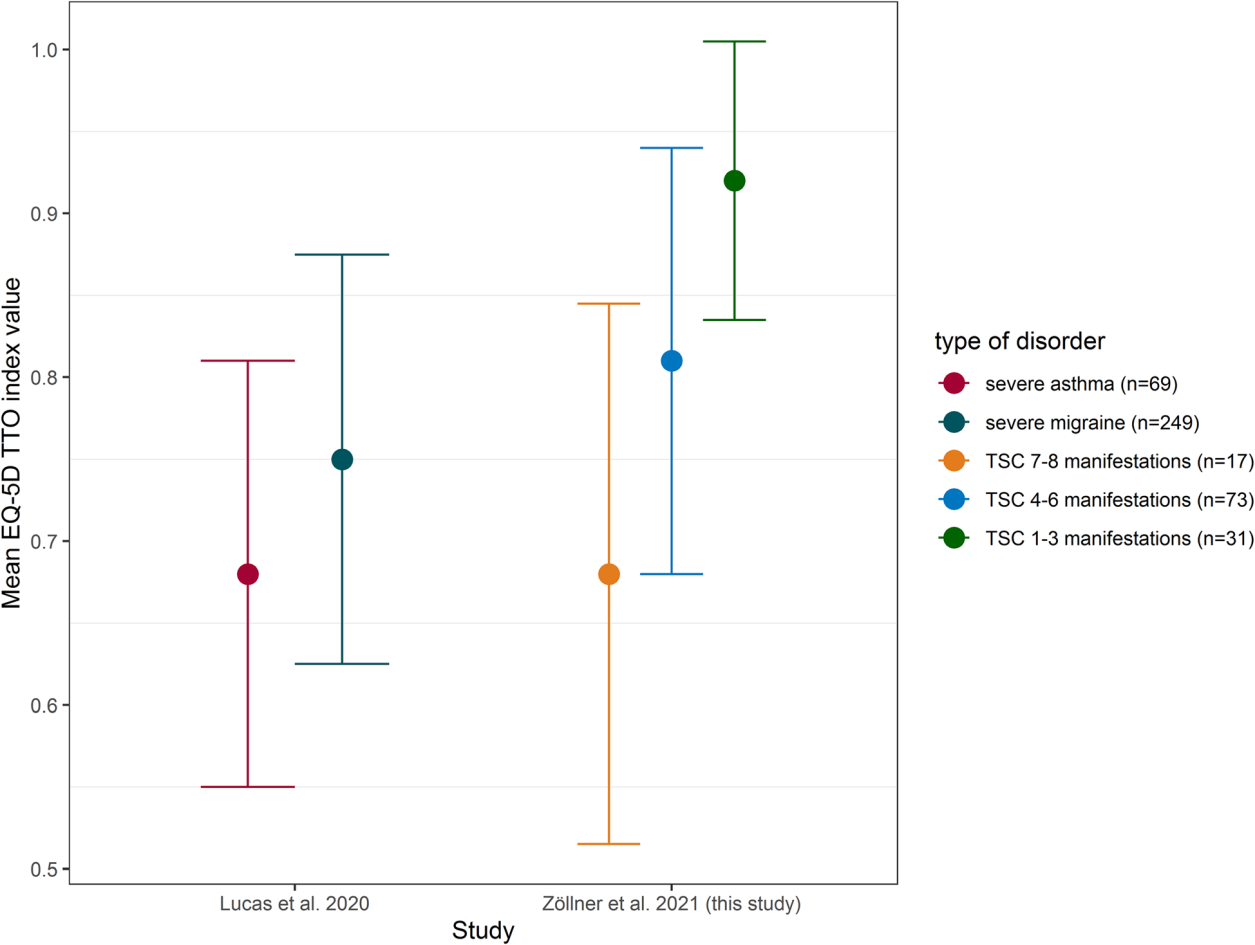
Generic quality of life (EQ-5D) among TSC patients compared with patients with other chronic disorders. Comparison of quality of life (QoL), as assessed by the EuroQoL-5 dimensions (EQ-5D), between a study (Lucas et al., [38]) including 69 patients with severe asthma and 249 patients with severe migraine and our cohort of tuberous sclerosis complex (TSC) individuals, stratified by the number of TSC manifestations. A large difference in QoL between lightly and more severely affected TSC individuals is evident, with more severely affected individuals exhibiting QoL similar to patients with other severe chronic disorders

Mildly affected (≤3 manifestations) individuals report a generic QoL of 80.9 (*SD* = 12.3, corresponding EQ-5D TTO index: 0.931, *SD* = 0.159), comparable to or even better than the overall German population with a single health issue (72.3 according to [37]). More severe manifestations were associated with significantly worse QoL (EQ-5D TTO index: 0.784, *SD* = 0.279), similar to those with severe chronic diseases, including severe episodic migraines with more than eight headache days per month (EQ-5D index: 0.77, *SD* = 0.24; Fig. 3) [38].

### Predictors of health-related quality of life

In multiple linear regression analysis, the overall LAEP score was an independent predictor of worse HRQoL, with each additional LAEP score point reducing overall QOLIE-31 by almost one point (B = − 0.906, 95% CI: − 1.192 to − 0.621, *p* < 0.001), with large effect size (*f*^*2*^ = 0.71). The presence of active epilepsy reduces the QOLIE-31 score by 12.6 points (B = − 12.608, 95% CI: − 18.234 to − 6.981, *p* < 0.001), with large effect size (*f*^*2*^ = 0.35). The presence of neuropsychiatric manifestations worsens QOLIE-31 by 10.4 points (B = − 10.401, 95% CI: − 17.034 to − 3.769, *p* = 0.003), with medium effect size (*f*^*2*^ = 0.173). The number of affected organs, employment, and grade of disability were not significant predictors in the final regression model, which accounted for 65% of HRQoL variance (*p* < 0.001; Table 5). The minimal clinically relevant difference for QOLIE-31 in TSC is approximately 11.1 points [39]; however, this was evaluated for longitudinal differences in a solely drug-refractory TSC-epilepsy cohort and is not directly applicable to our group.

**Table 5:** Multiple linear regression analyses evaluating potential TSC-related predictors of health-related quality of life (QOLIE-31 overall score)

	Regression coefficient ***B***	***SE B***	beta	***p***-value	Semi-partial correlation	Tolerance
LAEP total score	−0.906	0.143	−0.548	< 0.001	− 0.644 | *f*^*2*^ 0.709 – large effect	0.757
Active epilepsy (yes/no)	−12.608	2.810	−0.358	< 0.001	−0.511 | *f*^*2*^ 0.353 – large effect	0.885
Neuropsychiatric symptoms (yes/no)	−10.401	3.312	−0.293	0.003	−0.384 | *f*^*2*^ 0.173 – medium effect	0.644
Number of affected organs	1.234	1.066	0.102	0.252	0.152	0.726
Employment (yes/no)	2.457	2.951	0.070	0.409	0.110	0.800
Grade of disability (≤80/> 80)	−1.081	3.390	−0.030	0.751	−0.042	0.615
	Adjusted *R*^*2*^ = 0.65; *F* = 20.15, Sig. F. (*p* < 0.001)					

Variables are ordered according to the standardised B (beta) values, in descending order

*LAEP* Liverpool Adverse Events Profile*, SE* standard error. Effect sizes were calculated from the semi-partial correlation as *r*^*2*^_*part*_ / (1 − *r*^*2*^_*part*_). Tolerance was used as a measure of predictor multicollinearity, with tolerance values above a threshold of 0.25 indicating no collinearity

## Discussion

This detailed, multicentre QoL study included a large sample of 121 adult individuals with TSC within a single healthcare system and contributed important new information regarding the relationships between clinical and sociodemographic aspects and QoL among individuals with TSC.

Only a few TSC clinical aspects were associated with worse QoL. Specifically, neuropsychiatric manifestations (such as anxiety and depression) and active epilepsy (seizures within the preceding 12 months) reduced QoL. Neuropsychiatric manifestations in TSC are often severe and challenging to treat [5]. It is thus easily comprehensible that QoL among individuals who experience them is worse, however the magnitude of this effect and its persistence in the multivariable analysis was striking. The worsened QoL associated with active epilepsy may be attributable to the persistent burden and social and medical consequences of continuing seizures for the individual, as they are a constant reminder of TSC. By contrast, other manifestations, including renal AML, may only be intermittently burdensome or at later stages, with less effect on QoL.

A higher number of TSC manifestations was associated with reduced QoL, and QoL stratification according to manifestation number provided an insightful look at the range of QoL among individuals with TSC. Those with mild TSC reported QoL comparable to the overall German population, whereas those with multiple manifestations reported substantially worse QoL, similar to severe (more than 8 headache days/month, failure of ≥2 prophylactic medications) chronic migraine or uncontrolled asthma [38]. Therefore, TSC should not be interpreted as a disorder associated with a homogenous QoL simply due to its chronic nature: Distinct differences in QoL were notable, associated with disease burden and neuropsychiatric manifestations.

Therapy-related adverse events reduced QoL among TSC patients, and LAEP was a stronger predictor of reduced QoL in multivariate analysis than neuropsychiatric manifestations or active epilepsy. Although we cannot attribute adverse events to individual medications in this study, neither everolimus nor anti-seizure medication polytherapy [40, 41] was associated with worse QoL when examined individually, suggesting another possible interpretation of the LAEP results. LAEP scores are known to be influenced by the presence of anxiety and depression [42], which are among the most common neuropsychiatric manifestations in adults with TSC [43, 44] and were common in our cohort, with 40.5% affected according to the anxiety domain of the EQ-5D and a mean NDDI-E score of 12.4 (depression highly likely above 13 points). Thus, the LAEP may co-measure affective problems among individuals with TSC.

The perceived TSC-specific burden of illness, as assessed using a 20-item Likert scale, correlated highly with QoL (Fig. 1d), with higher disease burden values significantly associated with reduced QoL. Our scale was constructed as an exploratory tool and has not been previously validated; however, the strong correlation with QoL demonstrates conceptually that a relatively simple questionnaire can capture a broad range of potential burden of illness issues in TSC in a manner that is relevant for assessing QoL.

Although QoL has been used as an outcome variable for some TSC therapy studies (e.g. everolimus [45] and epilepsy surgery [46]), sparse literature addresses QoL in TSC overall. However, a range of studies has recently been published exploring this issue. Amin et al. [14] demonstrated that the psychosocial QoL domain is the most affected in those with TSC, and those with and without epilepsy have worse QoL than the general population. Both findings were supported by our study; the “usual activities” and “anxiety” domains of the EQ-5D were the most commonly affected, and QoL was worse regardless of TSC-associated epilepsy, although those with epilepsy showed comparatively worse QoL. Similar to Vergeer et al., who found worse QoL in those with severe impairment of daily functioning [16], indicators of worse daily functioning, including a higher grade of disability and unemployment, were associated with worse QoL in our study.

Quantitatively, generally higher HRQoL was observed in our cohort than among individuals with TSC from the TOSCA study [12]. However, the QOLIE-31 reporting among this comparator cohort was limited (*n* = 24), making comparisons prone to bias. Among the larger comparator registry patients reporting EQ-5D (*n* = 143), some categories demonstrated more frequent problems than our cohort, including the EQ-5D mobility (23.8% vs 12.4%) and pain (35% vs 27%) domains, whereas other problems were less frequent than in our cohort. A central issue associated with self-reported QoL assessments is the potential distortion towards a better QoL because more severely affected patients may be unable to complete the questionnaire. Compared with our results, a recent study demonstrated worse overall QoL among individuals with TSC but incorporated a high share of caregiver-reported outcomes [15]. Common QoL questionnaires, including the standard EQ-5D, were not constructed for by-proxy answering, which may introduce the underreporting of issues that rely on internal perception (e.g. pain).

The Epilepsy Stigma Scale was developed to detect stigma among epilepsy patients but features no questions specific to epilepsy. We found that a range of clinical TSC manifestations was associated with severe stigma. However two logically plausible clinical factors (number of TSC manifestations overall and presence of visible skin manifestations) were not associated with severe stigmatisation. On multiple regression, only the LAEP score remained an independent predictor of severe stigma. This may be due to individuals with more severe affective problems (using the LAEP as a proxy instrument) tending to feel more vulnerable and stigmatised. Taken together, these findings imply that affective issues have a stronger influence on feeling stigmatised than actual (potentially visible, and thus identifying) clinical TSC manifestations.

### Limitations

The evaluation of self-reported QoL and burden of illness measures risks under-capturing true QoL due to the exclusion of the most severely affected individuals. However, we found a significant QoL range within our cohort, demonstrating a wide range of participation. In addition, we identified a relevant ceiling effect in the EQ-5D results, indicating that caution remains necessary when using this instrument for clinically heterogeneous disorders such as TSC. An individual psychiatric examination would have provided further insight into the precise contribution of depression or other mental illness to quality of life than was possible in this questionnaire study. The additional survey of the Beck’s Depression Inventory would also have allowed a more precise differentiation.

## Conclusions

The severity of TSC-associated seizures, therapy-related adverse events, and neuropsychiatric manifestations, particularly anxiety and depression, appear to worsen quality of life among adults with TSC. Further research on quality of life among adult individuals with TSC should focus on differentiating the roles of therapy-related adverse events from pre-existing affective problems, such as anxiety and depression.

## Data Availability

The datasets analysed during the current study are available from the corresponding author on reasonable request.

## Abbreviations

AML: Angiomyolipoma
EQ-5D: EuroQoL-5 dimensions
HRQoL: Health-related quality of life
LAEP: Liverpool Adverse Events Profile
NDDI-E: Neurological Disorders Depression Inventory for Epilepsy
QoL: Quality of life
QOLIE-31: Quality of Life in Epilepsy Inventory-31 items
SEGA: Subependymal giant cell astrocytoma
TTO: Time-trade-off
TSC: Tuberous sclerosis complex
VAS: Visual analogue scale
